# Body image dissatisfaction and lower self-esteem as major predictors of poor sleep quality in gynecological cancer patients after surgery: cross-sectional study

**DOI:** 10.1186/s12905-021-01375-5

**Published:** 2021-06-03

**Authors:** Amina Aquil, Ouassil EL Kherchi, Naoual EL Azmaoui, Mustapha Mouallif, Maroua Guerroumi, Aziz Chokri, Arumugam R. Jayakumar, Abdellatif Benider, Abdeljalil Elgot

**Affiliations:** 1 Epidemiology and Biomedical Unit, Laboratory of Sciences and Health Technologies, Higher Institute of Health Sciences, Hassan First University of Settat, 26000 Settat, Morocco; 2 Institut des Sciences du Sport, Hassan First University of Settat, 26000 Settat, Morocco; 3 Neuropathology Research Unit, Miami VA Medical Center and Department of Obstetrics, Gynecology and Reproductive Sciences, University of Miami Miller School of Medicine, Miami, FL 33136 USA; 4 Ibn Rochd University Hospital Center, Mohammed VI Center for the Treatment of Cancers, Casablanca, Morocco; 5 Institut supérieur des sciences de la santé, Complexe Universitaire, route de Casablanca, B.P 555, Settat, Morocco

**Keywords:** Body image, Gynecological cancer, Self-esteem, Sleep quality

## Abstract

**Background:**

Sleep quality is among the indicators associated with the quality of life of patients with cancer. A multitude of factors may affect patient sleep quality and are considered as associated predictive factors. The aim of this study was to examine the predictors of poor sleep quality in Moroccan women with gynecological cancer after radical surgery.

**Methods:**

A cross-sectional study was carried out at the Oncology Department of the Ibn Rochd University Hospital, Casablanca (Morocco), on women who had undergone radical surgery for gynecological cancer (n = 100; mean age: 50.94 years). To assess sleep quality, symptoms of depression and anxiety, self-esteem and body image, the following translated and validated Arabic versions of the tools were used: Pittsburgh Sleep Quality Index (PSQI), Hospital Anxiety and Depression Scale, Rosenberg’s Self-Esteem Scale and Body Image Scale. To determine predictors of sleep quality, multiple linear and hierarchical regressions were used.

**Results:**

78% of participants were considered poor sleepers, most of them exhibited very poor subjective quality (53%), longer sleep onset latency (55%), short period of sleep (42%) and low rate of usual sleep efficiency (47%). 79% of these patients did not use sleep medication and 28% were in poor shape during the day. Waking up in the middle of the night or early in the morning and getting up to use the bathroom were the main reasons for poor sleep quality. Higher PSQI scores were positively correlated with higher scores of anxiety, depression, body image dissatisfaction and with lower self-esteem (*p* < 0.001). The medical coverage system, body image dissatisfaction and low self-esteem predicted poor sleep quality. After controlling for the socio-demographic variables (age and medical coverage system), higher body image dissatisfaction and lower self-esteem significantly predicted lower sleep quality.

**Conclusion:**

Body image dissatisfaction and lower self-esteem were positively linked to sleep disturbance in women with gynecological cancer after undergone radical surgery. These two predictors require systematic evaluation and adequate management to prevent sleep disorders and mental distress as well as improving the quality of life of these patients.

**Supplementary Information:**

The online version contains supplementary material available at 10.1186/s12905-021-01375-5.

## Background

The burden of cancer incidence is increasing worldwide and is regarded to be a global and serious public health problem [1]. Gynecological cancers were reported to be major sources of cancer related mortality in Moroccan women. According to Globocan 2018 statistics, cervical cancer is ranked second and ovarian cancer is ranked in the fifth position among cancers in Moroccan women [2].

Hysterectomy and or oophorectomy are known to be the main surgical treatments after the diagnosis of cancer of the cervix, uterus and ovary. In many cultures, besides breast, the uterus and ovaries were considered as symbols of femininity, fertility and especially motherhood. Indeed, the loss of these organs after surgery makes end to reproductive capacity and affects the ultimate goal of procreation in some countries [3, 4]. After undergoing radical surgery, gynecological cancer patients become vulnerable to several disorders impacting their quality of life, such as anxiety, depression, body image disorders and low self-esteem [5, 6].

Next, sleep quality is considered to be an important indicator of quality of life [7]. Therefore, many studies have reported that the prevalence of sleep disturbances in cancer patients varies between 20 and 87% [8–13], and the most observed sleep problems in cancer patients were insomnia (31%) and excessive sleepiness (28%) [14]. These patients suffer from greater sleep alterations, compared to the general population [15]. Among women with breast cancer, higher scores on depression and fatigue were associated with lower sleep quality [16]. Recently, higher hopelessness predicted higher sleep disturbance in women with breast cancer [9], while no such data were reported for women with gynecological cancer.

Thus, the management of sleep disturbances and the identification of its major predictors is crucial for patients with cancer, above as regards the time lapse between the diagnosis and the medical intervention, and as regards the post-operative life. To our knowledge, and to date, there is no published study specifically devoted in sleep disorders in North African women with gynecological cancer after radical surgery. Very few international studies have studied separately the association between self-esteem, anxiety, depression and sleep disorders in women with cancer. These studies showed that sleep disorders were significantly correlated with anxiety-depressive disorders and impaired self-esteem [7, 8, 17–19]. However, these studies were not specific to women with gynecological cancer who have undergone radical surgery. Further, these studies did not address the relationship between self-esteem, body image disturbances and sleep disturbances in women gynecological cancer after radical surgery. However, to answer to these questions is important for the following reasons: exploring the predictors of sleep quality, a prognostic indicator of survival in women with cancer, and managing them improves the quality of life of women with gynecological cancer [7].

The purpose of this study was to investigate the prevalence of sleep disorders in a sample of post-operative gynecological cancer patients in Morocco, and in particular to determine the predictors of these disorders in this category of patients; indeed, the ultimate goal of our study is to help nurses and oncology professionals to assess and manage sleep disorders and to improve the quality of life of these patients.

## Methods

### Design, setting, and participants

The present descriptive and cross-sectional analysis was carried out over a period of 10 months, in the Mohammed VI Center for the Treatment of Cancers, Ibn Rochd University Hospital in Casablanca, Morocco. A total of 104 women, who had undergone radical surgery for gynecological cancer, were invited to take part of the study, when they were at the oncology department for follow-up counseling. Of the 104 individuals approached, 100 agreed to participate. The inclusion criteria were: 1. 18 years old or older; 2. confirmed diagnosis of primary gynecological cancers (cervical, body uterus or ovarian), 3. T1–T3 tumor stages, followed by hysterectomy/oophorectomy for more than 3 weeks and not more than 6 months; 4. signed written informed consent (for unschooled patients a verbal consent followed by a fingerprint were used as a consent proof); 5. Willing and able comply with the study conditions. Exclusion criteria were: 1. not born in Morocco; 2. T4 tumor stage; 3. unable to understand and speak the Moroccan Arabic dialect; 4. Cognitive impairment or hearing loss; 5. history of any psychiatric disorder; 6. previous diagnosis of primary cancer in a different location.

### Procedure

Oncologists were randomly invited women who consulted for a check-up to take part of the study. The researcher verbally presented the study to the patients and woman who did not wish to participate were excluded. The researcher read each question and its answers, and then ticked the selected answer. The average time dedicated to fulfill the questionnaire was between 20 and 30 min. Patients were recruited consecutively until the sample size was sufficient. Socio-demographic and medical data were directly collected from patients and supplemented by medical records (Additional file 1).

### Measurements

#### Socio-demographic and medical characteristics

Sociodemographic characteristics were obtained through a questionnaire to ascertain the following: Age (years), marital status (married, single/divorced/widowed), having children (yes, no), medical coverage system (Ramed; The Medical Assistance Plan, known by the acronym RAMED, its aim is to guarantee the right to health care to economically disadvantaged people, CNOPS/CNSS; Medical coverage management bodies, it guarantees the right to health care to salaried employees), living area (rural, urban), educational level (none, compulsory school, higher educational degrees), working status (housewives/Retired, workers), socioeconomic level (poor, good). Clinical characteristics were obtained from participants and were completed with medical records. These included: Type of surgery (hysterectomy, oophorectomy), tumor stage (T1, T2, and T3), time since diagnosis (< 1 year, ≥ 1 year) and disease recurrence (yes, no).

#### Pittsburgh Sleep Quality Index (PSQI)

The Pittsburgh Sleep Quality Index (PSQI) developed in English [20], then translated and validated in Arabic [21], assesses sleep quality during the previous month. The scale contains 7 components (subjective quality of sleep, sleep latency, duration of sleep, usual sleep efficiency, sleep disturbances, use of sleeping pills and daytime dysfunction) encompassing 19 questions of self-evaluation. The score for each component can range from 0 (no difficulty) to 3 (severe difficulty). The sum of the 7 components gives the total PSQI score (between 0 and 21 points). The score 0 indicates that there is no difficulty while 21 indicates major difficulties. Subjects with a PSQI score greater than 5 are poor sleepers while those with a PSQI score equal to or less than 5 are good sleepers. The Cronbach's alpha for the PSQI used in this study is 0.89, which shows good validity (Additional files 1, 2).

#### Hospital Anxiety and Depression Scale (HADS)

The Hospital Anxiety and Depression Scale (HADS), developed in English [22], then translated and validated in Arabic [23], assesses the dimensions of anxiety and depression in non-psychiatric populations and largely used in psycho-oncology research. The HADS consists of two sub-scales of 7 items each, which give two scores measuring, the first one for anxiety HAD-A, and the second one for depression HAD-D. Patients can be divided into two groups: Patients with a total score less than 11 for HADS-A or HADS-D did not have proven anxiety or depressive disorders, respectively. Patients with a total score of 11 or higher for HADS-A or HADS-D were diagnosed with clinical anxiety or depression, respectively. The Cronbach’ alpha for the HADS scale used in this study is 0.98 which exhibiting a very good validity.

#### Rosenberg’s Self-Esteem Scale (RSES)

Self-esteem was evaluated with Rosenberg’s self-esteem scale (RSES). It comprises 10-item with four-point intensity scale; the total score is ranging from 0 to 30 points. Higher scores indicate better self-esteem [24]. Internal consistency in a number of studies on Arab populations has been good [25–27]. Cronbach's alpha for this study is 0.87 which demonstrated a good reliability of our questionnaire (Additional file 3).

#### Body Image Scale (BIS)

In the current study, the body image dissatisfaction regarding gynecological cancer patients was assisted using the Body Image Scale (BIS) developed by Hopwood and his collaborators in 2001 [28]. This scale includes 10 items developed to evaluate briefly and consistently the affective, the behavioral and the cognitive aspects of body image in cancer patients who are undergoing appearance changes. It reflects also the impact of cancer treatment, including surgery, on body image of gynecological cancer patients. The BIS components score ranges from 0 (not at all) to 3 (very much) and the BIS final score (the sum of the 10 items subscales) is ranging from 0 (body image satisfaction) to 30 (strong body image dissatisfaction). The higher the score, the more the body image is altered. The threshold value was 10. The Cronbach’s alpha for this study is 0.98 which demonstrated a very a good reliability of the used questionnaire.

RSES and BIS were the subject of a translation from English to Arabic and back-translation by translators/linguists. Then, the translations were examined and merged in a single version validated by Moroccan universities experts in this field to ensure the accuracy of the translation and the validity of the content. Subsequently, a pilot study was conducted on a group of 30 cancer patients excluded from the study. No changes were required to the questionnaire based on the piloting. The questionnaires were adapted and the final version was used.

### Statistical analyses

Descriptive statistics were performed to describe socio-demographic and medical characteristics participants. Verification of normality and homogeneity of variances was carried out by a Shapiro–Wilks test. These conditions were not met in our study. As a result, Fisher’s exact bilateral test to compare percentages, and the Spearman rank correlation coefficients were calculated to study the correlation between two quantitative variables. The difference between the groups for a continuous variable was tested by the Mann–Whitney test (in the presence of two modalities) or the Kruskal–Wallis test (for comparison of several averages). To identify predictors of sleep quality, the multiple linear regression was used including the variables most correlated with the PSQI score. To determine the unique impact of mental health variables on sleep quality, three sets of predictors have been introduced in stages in a hierarchical multiple regression model; these sets of predictors were: demographic (age), social (medical coverage system) and mental (anxiety, depression, body image dissatisfaction, self-esteem). Demographic variables were included in the first step. The medical coverage system was related to the quality of sleep in our sample and was therefore included as a potential social confounding factor in the second step. In the third step, we entered the scores of anxiety, depression, dissatisfaction with body image and self-esteem.

All *p* values equal to or less than 0.05 were considered statistically significant. The statistical analysis was carried out using the SPSS® version 20.0 (IBM Corporation, Armonk NY, USA) for Windows®. Microsoft Excel 2010 was used for inserting figures.

### Results

The Socio-demographic and clinical characteristics of the participants were represented in the Table 1.

**Table 1:** Socio-demographic and clinical characteristics of the gynecological cancer sample (N = 100)

	Min	Max	Median (Q1–Q3)	Number (%)
Age	30	76	52.50 (40.25–60)	
*A. Socio-demographic characteristics*				
Marital status				
Married				39
Single/divorced/widow				61
Have children				
No				37
Yes				63
Medical coverage system				
RAMed				88
CNOPS/CNSS				12
Living area				
Rural				42
Urban				58
Educational level				
None				64
Compulsory school, higher educational degrees				36
Working status				
Housewives/retired				84
Workers				16
Socio economic status				
Poor				84
Good				16
*B. Clinical characteristics*				
Surgery				
Hysterectomy				75
Oophorectomy				25
Tumor stage				
T1				23
T2				8
T3				69
Time since diagnosis				
< 1 an				69
≥ 1 an				31
Antineoplastic treatment				
One treatment				66
Two treatment				33
Three treatment				1
Disease recurrence				
No				85
Yes				15
Anxiety (HADS-A)	0	21	13 (7–18)	
Depression (HADS-D)	0	21	12.5 (7–17.75)	
Body image (BID)	0	30	10 (4–24)	
Self esteem (RSES)	0	30	19 (10–25)	

*HADS-A* Hospital Anxiety and Depression Scale-Anxiety, *HADS-D* Hospital Anxiety and Depression Scale-Depression, *BID* Body image dissatisfaction, *RSE* Rosenberg's Self-Esteem Scale, *PSQI* Pittsburgh Sleep Quality Index, *Q1–Q3* interquartile interval

Sixty-six % of the patients reported symptoms of anxiety; 59% reported severe symptoms of depression, and 54% reported body image problems.

Thirty-nine % reported very poor subjective sleep quality, and 14% reported somewhat poor quality. For sleep onset latency, 35% needed more than one hour to fall asleep, and 20% needed between 31 and 60 min to fall asleep. The sleep duration did not exceed 5 h per night for 42% of the participants, with a median of 5 h per night (Table 2).

**Table 2:** Sleep characteristics of the gynecological cancer sample (N = 100)

General characteristics of sleep	Min	Max	Median (Q1–Q3)
Usual bedtime	21	24	23 (22–24)
Sleep latency (min)	5	180	60 (30–120)
Usual time of waking up	4	11	5 (5–7)
Hours for sleep of night	2	9	5 (3–6)
*PSQI components*			
Subjective sleep quality	0	3	2 (0–3)
Sleep latency	0	3	3 (1–3)
Sleep duration usual	0	3	2 (1–3)
sleep efficiency	0	3	2 (1–3)
Sleep disturbances	0	3	1 (0–2)
Use of sleep medications	0	3	0 (0–0)
Daytime dysfunction	0	3	2 (1–3)
PSQI overall score	1	20	12 (6–16)

*PSQI* Pittsburgh Sleep Quality Index, *Q1–Q3* interquartile interval

**Fig. 1:**
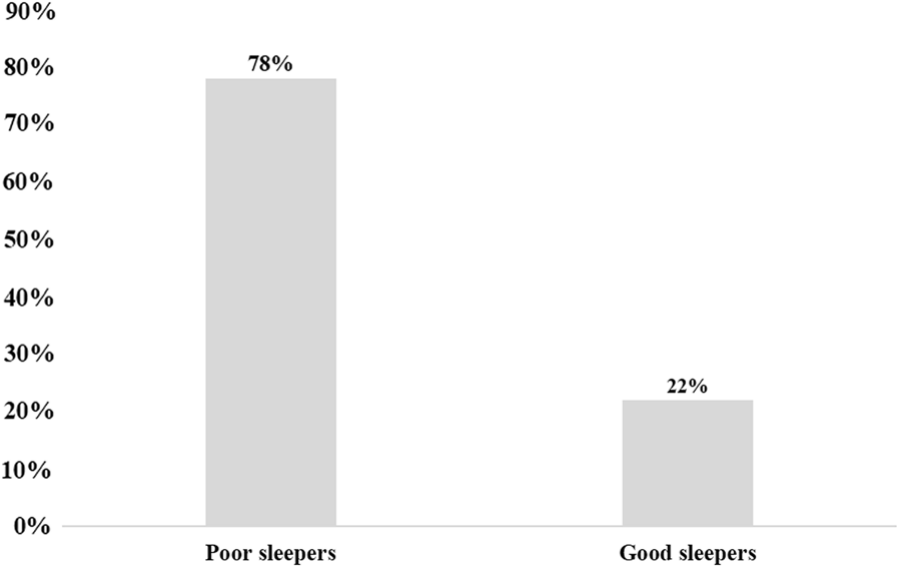
Prevalence of sleep disorders. According to a threshold value of 5 at the PSQI, 78% of gynecological cancer women were considered as poor sleepers

Forty-seven % of participants had usual sleep efficiency less than 65%, while the latter exceeded 85% in 18% of participants. The sleep disorders in the participants were explained by several reasons, mainly citing: waking up in the middle of the night or early in the morning (68%), getting up to use the bathroom (62%), having nightmares (42%), feeling hot (38%), having breathing problems (35%), having pain (26%), feeling cold (18%) and coughing and snoring loudly (16%). Only 21% of the participants claimed the use of sleep medication and 28% had daytime dysfunction. Finally, 78% of the participants were considered to be poor sleepers (Fig. 1).

Higher PSQI scores were significantly associated with younger age (*p* = 0.001, r = −0.32). PSQI scores significantly tended to be higher among unmarried women (single/divorced/widowed), those who had no children, those who had Ramed, those who worked, those who had lower economic status and women with time since diagnosis did not exceed 1 year (*p* < 0.05) (Table 3).

**Table 3:** Association between sleep quality, socio-demographic and clinical characteristics

	Median (Q1–Q3)	r	*p* value
*Socio-demographic characteristics*			
Sleep quality (PSQI)			
Age	52.50 (40.25–60)	**− 0.32**	**0.001**
Marital status			
Married	7 (4–15)		**0.006**
Single/divorced/widow	13 (7–17)		
Have children			
No	17 (12–17)		**0.04**
Yes	7 (4–14)		
Medical coverage system			
RAmed	12 (7–17)		**0.000**
CNOPS/CNSS	4 (2–7)		
Living area			
Rural	13.5 (7–17)		0.102
Urban	11.5 (4–16)		
Educational level			
None	12 (6–16)		0.759
Compulsory school, higher educational degrees	12 (4.75–16.75)		
Working status			
Housewives/retired	10.5 (6–16)		**0.001**
Workers	17 (13–17)		
Socio economic status			
Poor	12 (7–17)		**0.001**
Good	4 (3–7)		
*Clinical characteristics*			
Surgery			
Hysterectomy	11 (6–16)		0.111
Oophorectomy	15 (7–17)		
Tumor stage			
T1	7 (5–16)		0.689
T2	14.5 (4–15.75)		
T3	12 (7–17)		
Time since diagnosis			
< 1 an	13 (7–17)		**0.030**
≥ 1 an	7 (4–15)		
Antineoplastic treatment			
One treatment	7 (4–15)		0.299
Two treatment	8 (4–17)		
Three treatment	2 (2–2)		
Disease recurrence			
No	12 (6–16)		0.476
Yes	15 (7–17)		

Values in bold: Significant association (*p* value < 0.05)

*PSQI* Pittsburgh Sleep Quality Index, *Q1–Q3* interquartile interval

*p* value obtained with Mann–Whitney U-test and Kruskal–Wallis test

Higher PSQI scores were positively correlated with higher symptoms of anxiety (*p* < 0.001), depression (*p* < 0.001), and body image dissatisfaction (*p* < 0.001), and negatively correlated with lower self-esteem (*p* < 0.001) (Table 4).

**Table 4:** Intercorrelations between the various variables of the study

	1	2	3	4	5	6	7	8	9	10	11	12
1. Subjective sleep quality	–											
2. Sleep latency	0.770**	–										
3. Sleep duration Usual	0.664**	0.650**	–									
4. Sleep efficiency	0.635**	0.598**	0.703**	–								
5. Sleep disturbances	0.747**	0.579**	0.552**	0.560**	–							
6. Use of sleep medications	0.122	− 0.032	− 0.039	− 0.030	0.331*	–						
7. Daytime dysfunction	0.747**	0.660**	0.522**	0.486**	0.530**	0.034	–					
8. PSQI overall score	0.905**	0.841**	0.781**	0.765**	0.810**	0.170	0.776**	–				
9. Anxiety (HADS-A)	0.656**	0.529**	0.365*	0.329*	0.518**	0.002	0.489**	0.584**	–			
10. Depression (HADS-D)	0.688**	0.496**	0.333*	0.313	0.581**	0.151	0.439**	0.596**	0.954**	–		
11. Body image (BID)	0.541**	0.260	0.296	0.358*	0.425**	0.051	0.367*	0.448**	0.396*	0.489**	–	
12. Self esteem (RSES)	− 0.702**	− 0.497**	− 0.334*	− 0.323*	− 0.519**	− 0.116	− 0.384*	− 0.568**	− 0.865**	− 0.913**	− 0.423**	–

*HADS-A* Hospital Anxiety and Depression Scale-Anxiety, *HADS-D* Hospital Anxiety and Depression Scale-Depression, *BID* body image dissatisfaction, *RSE* Rosenberg's Self-Esteem Scale, *PSQI* Pittsburgh Sleep Quality Index

*p < 0.05; **p < 0.001. Spearman's rank correlation coefficient

The main predictors of sleep quality according to multivariate analysis were: medical coverage system (*p* = 0.024), body image dissatisfaction (*p* = 0.009) and self-esteem (0.035) (Table 5).

**Table 5:** Results of the multivariate analysis predicting sleep disorders

	Sleep quality (PSQI)			
	B	CI 95%	t	*p* value
Age	0.107	(− 0.03; 0.13)	1.24	0.21
Marital status	− 0.015	(− 2.11; 1.77)	− 0.17	0.86
Have children	− 0.15	(− 3.85; 0.38)	− 1.63	0.11
Medical coverage system	− 0.20	(− 6.48; − 0.46)	− 2.29	0.024
Working status	0.025	(− 1.99; 2.75)	0.32	0.75
Socio-economic status	0.007	(− 2.79; 3.01)	0.07	0.94
Time since diagnosis	− 0.12	(− 3.23; 0.22)	− 1.72	0.08
Anxiety (HADS-A)	− 0.07	(− 0.56; 0.41)	− 0.30	0.76
Depression (HADS-D)	0.03	(− 0.47; 0.53)	0.12	0.90
Body image dissatisfaction (BID)	0.33	(0.04; 0.31)	2.66	0.009
Self-esteem (RSES)	− 0.39	(− 0.40; − 0.01)	− 2.14	0.035

*B* standardized regression coefficient, *CI 95%* confidence interval 95%, *p* degree of significance, *HADS-A* Hospital Anxiety and Depression Scale-Anxiety, *HADS-D* Hospital Anxiety and Depression Scale-Depression, *BID* body image dissatisfaction, *RSE* Rosenberg's Self-Esteem Scale, *PSQI* Pittsburgh Sleep Quality Index

According to the hierarchical regression analysis correlating the PSQI scores to the mental distress variables, after checking the key assumptions of the multiple regression (the linear relationship between independent variables and the dependent variable, the normality of residuals, the uncorrelated residuals, as well as the homoscedasticity) and after controlling for socio-demographic variables (age and medical coverage system), greater dissatisfaction with body image and lower self-esteem contributed significantly to the quality of sleep (Table 6).

**Table 6:** Hierarchical multiple regression model correlating sleep quality to psychological variables

Variables	Sleep quality (PSQI)											
	Step 1				Step 2				Step 3			
	B	CI 95%	r	*p*	B	CI 95%	r	*p*	B	CI 95%	r	*p*
AGE	− 0.28	− 0.23; − 0.04	− 0.28	0.004	− 0.26	(− 0.22; − 0.03)	− 0.27	0.005	0.09	(− 0.02; 0.12)	0.13	0.21
Medical coverage system	–	–	–	–	–0.34	(− 9.01; − 2.74)	− 0.35	0.000	− 0.19	(− 5.86; − 0.96)	− 0.27	0.007
Anxiety (HADS-A)	–	–	–	–	–	–	–	–	0.08	(− 0.36; 0.53)	0.03	0.71
Depression (HADS-D)	–	–	–	–	–	–	–	–	− 0.16	(− 0.63; 0.32)	− 0.06	0.15
Body image (BID)	–	–	–	–	–	–	–	–	0.44	(0.12; 0.35)	0.38	**0.000**
Self esteem (RSES)	–	–	–	–	–	–	–	–	− 0.42	(− 0.41; − 0.03)	− 0.24	**0.019**

Values in bold: Significant association (*p* value < 0.05)

*B* standardized beta coefficient, *CI 95%* 95% confidence interval, *r* Spearman’s rank correlation coefficient, *HADS-A* Hospital Anxiety and Depression Scale-Anxiety, *HADS-D* Hospital Anxiety and Depression Scale-Depression, *BID* body image dissatisfaction, *RSE* Rosenberg's Self-Esteem Scale, *PSQI* Pittsburgh Sleep Quality Index

## Discussion

The current study had three main objectives; determine the prevalence of sleep disturbances in a sample of women with gynecological cancer after radical surgery, examine the predictors of impaired sleep quality and look for the relationship between anxiety, depression, body image disturbances, self-esteem and sleep disturbances in these patients.

The key findings of this study were that body image dissatisfaction and lower self-esteem were positively linked to sleep disturbance in women with gynecological cancer after undergone radical surgery. The present pattern of results adds to the current literature in an important way, because very few international studies have studied separately the association between self-esteem, anxiety, depression, body image disorders and sleep disorders in women with gynecological cancer after surgery. Results are of clinical and practical importance because these two predictors require systematic evaluation and adequate management to prevent sleep disorders and mental distress as well as improving the quality of life of these patients.

In this study, the vast majority of participants (78%) were considered poor sleepers. This result is consistent with data of a descriptive study conducted on women with breast cancer and gynecological cancer, for which the PSQI indicated that 80% of patients had sleep disorders during their stay at the hospital [29]. The components of PSQI show that most of the patients exhibited very poor subjective quality, long sleep onset latency, a short period of sleep and low rate of usual sleep efficiency. Moreover, the vast majority of these patients was in poor shape during the day and did not use sleep medication. The reasons which are basically responsible for hindering the quality of sleep and mostly cited by the patients were especially: waking up in the middle of the night or early in the morning and getting up to use the bathroom. These results are in the same line with a recent Tunisian study which assessed sleep disorders in patients with breast cancer after surgery [9].

In the present study, age was a factor associated with sleep disturbances, this factor was also cited by other study seeking predictors of poor sleep quality in patients with cervical cancer during adjuvant therapy [17]. In contrast, there was no association between age and sleep disorders in other study evaluating sleep quality in American women with ovarian cancer [7]. Marital status and having children were also among factors associated with the quality of sleep in women with gynecological cancer, this finding is in the same direction of another recent study claiming that married cancer women and those with more children had less sleep problems [9]. Moreover, the socioeconomic status, working status and medical coverage system in our participants were strongly associated with sleep quality. According to our data, poor patients suffered from various sleep disorders. This finding is in agreement with others from the literature suggesting that low socioeconomic status is a predictor of poor sleep quality in other types of cancer patients rather than gynecological ones [30]. In addition, our study showed that sleep quality is linked with time since diagnosis. This finding is inconsistent with a study showing that there was no correlation between PSQI and time since diagnosis in ovarian cancer patients [7].

This study showed that, in gynecological cancer patients who underwent a hysterectomy/ oophorectomy, the PSQI scores were positively correlated with anxiety and depression. Previous studies found a link between poor sleep quality and both anxiety and depressive disorders in cancer patients [7, 8, 17, 18]. It has been reported also that, in cancer patients, the level of emotional distress was significantly associated with the levels of self-esteem [31]. Regarding the relationship between self-esteem and sleep quality, it is revealed according to the hierarchical regression model that self-esteem constitutes one of the main predictors of sleep quality in our study. This result is consistent with a Chinese study showing a strong association between self-stigma and impaired sleep quality in cancer patients [19].

Body image is the representation of the self that is born “at the crossroads of the body and the psychic; it covers the permanence of the self in space, time and in relations to the world” [32]. Studies have suggested that cancer causes a huge alterations on body image [33]. Recent studies suggested that body changes caused by cancer or related surgery induced significant impairments not only on body image dissatisfaction but it seems to be a mental distress source [34]. In addition, body image dissatisfaction was cited in the literature as a predictor of quality of life of cancer patients [35]. Taking into account confounding factors, it turned out that disturbed body image is a main predictor of poor sleep quality in patients with gynecological cancer receiving radical surgery. Along the same lines of this result, a recent cross-sectional study found that body image was the main psychological variable exhibiting a strong impact on the quality of sleep of women with polycystic ovary syndrome [36].

To improve mental health status and quality of life of women with gynecological cancer after surgery, health decision-makers are being called upon to adopt new startups such as the ERAS protocol, a multi-modal, multidisciplinary approach to perioperative care, which has resulted in substantial improvements in clinical outcomes in quality of life, sleep dimensions, depression, distress and vitality after laparoscopic hysterectomy [37–39].

Given the results presented in this article, it is important to recognize several limitations. On the one hand, our sample may not be representative of the general population of Moroccan women with gynecological cancer due to the recruitment of participants in a single hospital. On the other hand, our study is cross-sectional; patients were assessed at a specific point in their follow-up. The study of the evolution of sleep disorders during the different stages of the disease evolution would have led to a better understanding of these disorders. However, this measure was difficult to achieve in the face of overloaded oncology visits and the inaccessibility of spaces that can ensure the privacy of participants during sleep quality assessment. However, our study is the first in Morocco and elsewhere, in which the effect of dissatisfaction with body image on the quality of sleep in patients with gynecological cancer is evaluated after radical surgery. In addition, our work is conducted at the university hospital center which welcomes patients coming from both urban and rural sector of the Casablanca-Settat region, the country's economic capital.

## Conclusion

Overall, our study showed high prevalence of sleep disorders in Moroccan women with gynecological cancers treated with radical surgery. Predictors of sleep quality included socio-demographic factors (age and system of medical coverage) as well as mental factors (anxiety, depression, body image disturbances and self-esteem). To ensure adequate psychotherapeutic care for these patients, systematic screening for sleep disorders and their predictors is necessary. Oncology health professionals and health decision makers are called upon to develop psychological strategies and integrate interventions that can help these patients gain their self-confidence and strengthen their self-esteem; these types of psychological care will undoubtedly improve the quality of life of patients with gynecological cancer and increase their life expectancy.

## Supplementary Information

**Additional file 1.** Mental health assessment in women with gynecological cancer.

**Additional file 2.** Informed consent of schooled patients.

**Additional file 3.** Informed consent of unschooled patients.

## Data Availability

The datasets used and/or analyzed during the current study are available from the corresponding author on reasonable request.

## Abbreviations

BIS: Body Image Scale
CNOPS: National Fund for Social Welfare Organizations
CNSS: National Social Security Fund
ERAS: Enhanced recovery after surgery
HADS: Hospital Anxiety and Depression Scale
HADS-A: Hospital Anxiety and Depression Scale-Anxiety
HADS-D: Hospital Anxiety and Depression Scale-Anxiety–Depression
RAMed: Medical Assistance Plan
RSES: Rosenberg’s Self-Esteem Scale
