# Supplementary material for: Body image dissatisfaction and lower self-esteem as major predictors of poor sleep quality in gynecological cancer patients after surgery: cross-sectional study
Source: BMC Womens Health. 2021 Jun 3;21:229. doi: 10.1186/s12905-021-01375-5 (PMC8173912; doi:10.1186/s12905-021-01375-5)
Supplement: Supplementary file 1 — Additional file 1. Mental health assessment in women with gynecological cancer. [file 12905_2021_1375_MOESM1_ESM.pdf]

جامعة الحسن الأول
UNIVERSITÉ HASSAN 1er

المعهد العالي لعلوم الصحة
INSTITUT SUPÉRIEUR
DES SCIENCES DE LA SANTÉ

Mental health assessment in women with gynecological cancer

• Thank you for accepting participation in this study which is part of scientific research and which aims to assess the state of mental health of women with gynecological cancer.

• This study guarantees the anonymity of participants and the information collected will be used for scientific research and public interest purposes. Please answer all questions.

I- Socio-demographic and clinical characteristics of participants:

1. Age:……
2. Marital status: 1-Married 2-Single 3-Divorced 4-Widow
3. Have children: 1-No 2-Yes
4. Medical coverage system: 1-Neither 2-RAMed 3-CNOPS/CNSS
5. Living area: 1-Rural 2-Urbain
6. Educational level: 1-None 2-Basic 3-Higher
7. Working status: 1-Houswife 2-Working 3-Retired
8. Socio economic status: 1-Poor 2-Good
9. Surgery: 1-Hysterectomy 2-Oophorectomy
10. Tumor stage: 1-T1 2-T2 3-T3
11. Time since diagnosis : 1- <1 an -≥1 an
12. Antineoplastic treatment:……….
13. Disease recurrence: 1-No 2-Yes

II- Hospital Anxiety and Depression Scale (HADS)

Tick the box beside the reply that is closest to how you have been feeling in the past week.
Don't take too long over you replies: your immediate is best.

D	A		D	A	
		**I feel tense or 'wound up':**			**I feel as if I am slowed down:**
	3	Most of the time	3		Nearly all the time
	2	A lot of the time	2		Very often
	1	From time to time, occasionally	1		Sometimes
	0	Not at all	0		Not at all
		**I still enjoy the things I used to enjoy:**			**I get a sort of frightened feeling like 'butterflies' in the stomach:**
0		Definitely as much		0	Not at all
1		Not quite so much		1	Occasionally
2		Only a little		2	Quite Often
3		Hardly at all		3	Very Often
		**I get a sort of frightened feeling asif something awful is about to happen:**			**I have lost interest in my appearance:**
	3	Very definitely and quite badly	3		
	2	Yes, but not too badly	2		Definitely
	1	A little, but it doesn't worry me	1		I don't take as much care as I should I
	0	Not at all	0		may not take quite as much care
					I take just as much care as ever
		**I can laugh and see the funny side of things:**			**I feel restless as I have to be on the move:**
				3	Very much indeed
0		As much as I always could		2	Quite a lot
1		Not quite so much now		1	Not very much
2		Definitely not so much now		0	Not at all
3		Not at all			**I look forward with enjoyment to things:**
		**Worrying thoughts go through my mind:**			As much as I ever did Rather less than I used to Definitely
	3	A great deal of the time	0		less than I used to Hardly at all
	2	A lot of the time	1		
	1	From time to time, but not too often	2		
	0	Only occasionally	3		
		**I feel cheerful:**			**I get sudden feelings of panic:**
3		Not at all		3	Very often indeed
2		Not often		2	Quite often
1		Sometimes		1	Not very often
0		Most of the time		0	Not at all
		**I can sit at ease and feel relaxed:**			**I can enjoy a good book or radio or TV program:**
	0	Definitely	0		Often
	1	Usually	1		Sometimes
	2	Not Often	2		Not often
	3	Not at all	3		Very seldom

III- Body image satisfaction

Tick the box beside the reply that is closest to how you have been feeling in the past week. Don't take too long over you replies: your immediate is best.

	Not at al	A little	Quite a bit	Very much
1.Have you been feeling self-conscious about your appearance ?				
2.Have you felt less physically attractive as a result of your disease or treatment ?				
3.Have you been dissatisfied with your appearance when dressed?				
4.Have you been feeling less feminine/masculine as a result of your disease or treatment ?				
5.Did you and it difficult to look at yourself naked?				
6.Have you been feeling less sexually attractive as a result of your disease or treatment?				
7. Did you avoid people because of the way you felt about your appearance?				
8. Have you been feeling the treatment has left your body less whole?				
9.Have you felt dissatisfied with your body ?				
10.Have you been dissatisfied with the appearance of your scar ?				

IV- Rosenberg self-esteem scale:

Below is a list of statements dealing with your general feelings about yourself. Please indicate how strongly you agree or disagree with each statement.

	Strongly Agree	Agree	Disagree	Strongly Disagree
1. On the whole, I am satisfied with myself.				
2. At times I think I am no good at all.				
3. I feel that I have a number of good qualities.				
4. I am able to do things as well as most other people.				
5. I feel I do not have much to be proud of.				
6. I certainly feel useless at times.				
7. I feel that I'm a person of worth, at least on an equal plane with others.				
8. I wish I could have more respect for myself.				
9. All in all, I am inclined to feel that I am a failure.				
10. I take a positive attitude toward myself.				

V- Pittsburgh Sleep Quality Index (PSQI)

The following questions relate to your usual sleep habits during the past month only. Your answers should indicate the most accurate reply for the majority of days and nights in the past month. Please answer all questions.

During the past month,

1. When have you usually gone to bed? ______________
2. How long (in minutes) has it taken you to fall asleep each night? ______________
3. What time have you usually gotten up in the morning? ______________
4. A. How many hours of actual sleep did you get at night? ______________
   B. How many hours were you in bed? ______________

5. During the past month, how often have you had trouble sleeping	Not during more the past month (0)	Less than once a week (1)	Once or twice a week (2)	Three or times a week (3)
A. Cannot get to sleep within 30 minutes				
B. Wake up in the middle of the night or early morning				
C. Have to get up to use the bathroom				
D. Cannot breathe comfortably				
E. Cough or snore loudly				
F. Feel too cold				
G. Feel too hot				
H. Have bad dreams				
I. Have pain				
J. Other reason (s), please describe, including how often you have had trouble sleeping because of this				
6. During the past month, how often have you taken medicine (prescribed or "over the counter") to help you sleep?				
7. During the past month, how often have you had trouble staying awake while driving, eating meals, or engaging in social activity?				
8. During the past month, how much of a problem has it been for you to keep up enthusiasm to get things done?				
9. During the past month, how would you rate your sleep quality overall?	Very good good (0)	Fairly (1)	Fairly bad (2)	Very bad (3)
