# Supplementary figures and images for: Body image dissatisfaction and lower self-esteem as major predictors of poor sleep quality in gynecological cancer patients after surgery: cross-sectional study
Source: BMC Womens Health. 2021 Jun 3;21:229. doi: 10.1186/s12905-021-01375-5 (PMC8173912; doi:10.1186/s12905-021-01375-5)

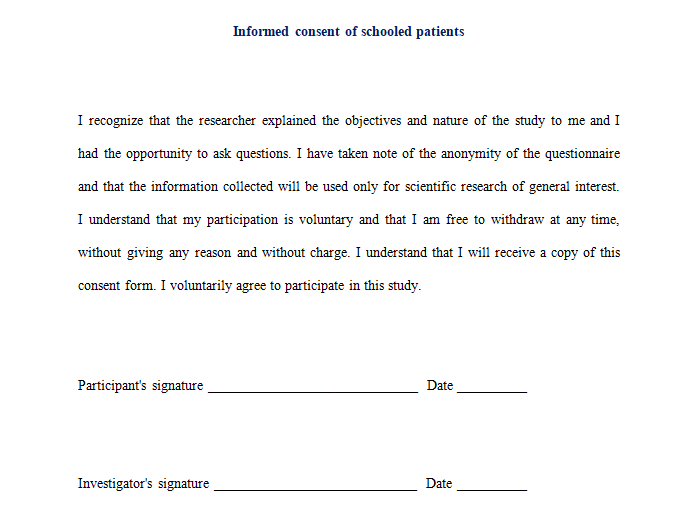

Supplement: Supplementary file 2 — Additional file 2. Informed consent of schooled patients. [file 12905_2021_1375_MOESM2_ESM.png]

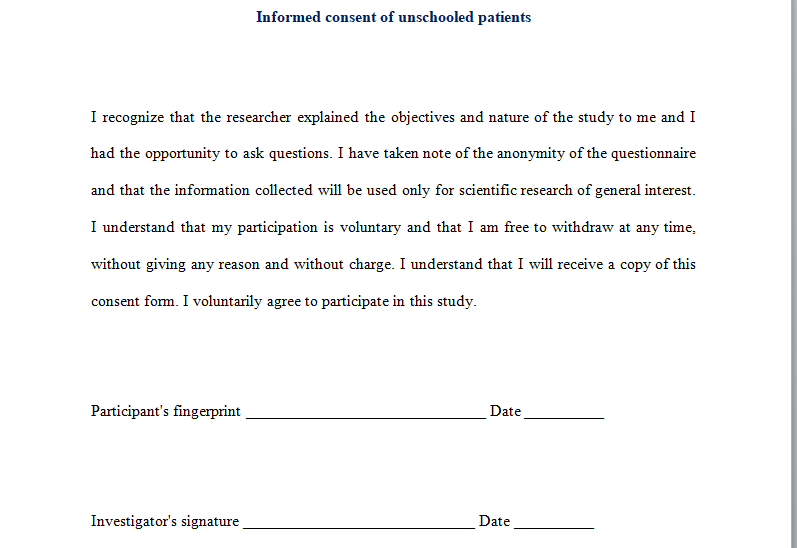

Supplement: Supplementary file 3 — Additional file 3. Informed consent of unschooled patients. [file 12905_2021_1375_MOESM3_ESM.png]
